# Effect of Repeated Anthelminthic Treatment on Malaria in School Children in Kenya: A Randomized, Open-Label, Equivalence Trial

**DOI:** 10.1093/infdis/jiv382

**Published:** 2015-07-13

**Authors:** Stella Kepha, Fred Nuwaha, Birgit Nikolay, Paul Gichuki, Charles S. Mwandawiro, Pauline N. Mwinzi, Maurice R. Odiere, Tansy Edwards, Elizabeth Allen, Simon J. Brooker

**Affiliations:** 1 School of Public Health, Makerere University College of Health Sciences, Kampala, Uganda; 2 London School of Hygiene and Tropical Medicine, United Kingdom; 3 Eastern and Southern Africa Centre of International Parasite Control, Kenya Medical Research Institute (KEMRI); 4 KEMRI–Wellcome Trust Research Programme, Nairobi; 5 Neglected Tropical Diseases Research Unit, Center for Global Health Research, KEMRI, Kisumu, Kenya

**Keywords:** malaria, helminths, deworming, school children, Kenya

## Abstract

***Background.*** School children living in the tropics are often concurrently infected with plasmodium and helminth parasites. It has been hypothesized that immune responses evoked by helminths may modify malaria-specific immune responses and increase the risk of malaria.

***Methods.*** We performed a randomized, open-label, equivalence trial among 2436 school children in western Kenya. Eligible children were randomized to receive either 4 repeated doses or a single dose of albendazole and were followed up during 13 months to assess the incidence of clinical malaria. Secondary outcomes were *Plasmodium* prevalence and density, assessed by repeat cross-sectional surveys over 15 months. Analysis was conducted on an intention-to-treat basis with a prespecified equivalence range of 20%.

***Results.*** During 13 months of follow-up, the incidence rate of malaria was 0.27 episodes/person-year in the repeated treatment group and 0.26 episodes/person-year in the annual treatment group (incidence difference, 0.01; 95% confidence interval, −.03 to .06). The prevalence and density of malaria parasitemia did not differ by treatment group at any of the cross-sectional surveys.

***Conclusions.*** Our findings suggest that repeated deworming does not alter risks of clinical malaria or malaria parasitemia among school children and that school-based deworming in Africa may have no adverse consequences for malaria.

***Clinical Trials Registration.*** NCT01658774.

Helminths and *Plasmodium* organisms are some of the most common parasites infecting humans worldwide [1, 2]. Their large-scale distributions are delineated by climatic factors, principally temperature and humidity, and modified by socioeconomic factors [1, 3]. The overlapping distributions of helminth and plasmodia species mean that coinfection with both types of parasites is common [4], with school-aged children at greatest risk [5, 6]. Helminth species elicit a strong immune response among hosts [7], and it has been hypothesized that this may influence, either positively or negatively, human immunity to malaria parasites and hence susceptibility to clinical malaria [8, 9]. However, previous studies have been typically cross-sectional and performed in single populations, and they have produced conflicting results [10–15].

A few randomized longitudinal studies have to date investigated interactions between worm and *Plasmodium* species. In Madagascar, bimonthly treatment with the anthelmintic levamisole had no effect on *Plasmodium falciparum* parasite density among children aged <5 years but, among children aged ≥15 years, resulted in a significant increase in parasitemia, compared with untreated controls [16]. A trial among Nigerian children aged 12–59 months found that the *Plasmodium* prevalence or density did not differ among those who received 4 monthly albendazole treatments, compared with children who received a placebo [17]. However, these trials had a small sample size, had inadequate follow-up, or used a drug (levamisole) that elicits an immune response. Recently, a cluster-randomized trial in Indonesia evaluated the impact of albendazole treatment received every 3 months for 21 months among children aged 5–14 years and reported a transient increase in malaria parasitemia at 6 months among older children but no significant impact at the end of the trial [18]. This latter study provides the strongest evidence to date that intensive deworming does not alter the risk of malaria among school-aged children living in Asia. A more recent individual randomized trial in northwestern Tanzania found that repeated treatment against schistosomes (using praziquantel) and soil-transmitted helminths (STH; using albendazole) did not alter the risk of clinical malaria or parasitemia, compared with annual treatment [19]; however the combined use of praziquantel and albendazole may have masked the effect of each treatment.

We present results from an individual randomized, open-label trial evaluating the impact of repeated (every 4 months) anthelmintic treatment with albendazole on clinical malaria and malaria parasitemia among school children in an area where only STH species are endemic. Our hypothesis was that, although helminths elicit strong immune responses, repeated anthelmintic treatment does not decrease or increase the risk of clinical malaria or malaria parasitemia, compared with annual treatment. Thus, we tested the hypothesis of no difference (equivalence) between the 2 treatment groups.

## MATERIALS AND METHODS

Reporting of the current trial is in accordance with the checklist of the extension of the CONSORT statement for noninferiority and equivalence randomized trials [20] (Supplementary Materials).

### Study Area and Population

The study was conducted between January 2013 and September 2014 in Bumula District, Bungoma County, western Kenya. The population of the area consists of indigenous Bukusu people and mainly Luhya who settled in recent years. The economy is primarily rural subsistence agriculture, with some families growing sugar cane as a cash crop. Cattle and sheep are commonly kept. Malaria transmission is intense and perennial, with 2 seasonal peaks (May–August and November–December). Most malaria is caused by *P. falciparum*, with recent survey data indicating a *P. falciparum* prevalence of 21.6% among school children [21]. Historically, helminth infections have been highly prevalent (89.6%) in the area [22, 23], but recent improvements in socioeconomic status and access to water and sanitation have reduced infection levels [24]. Recent data indicate that 25.1% of school children are infected with *Ascaris lumbricoides* and/or hookworm [25]. As part of the national school-based deworming program launched in 2009, all school children in the area were treated with 400 mg of albendazole in June 2013.

### Study Design

The study was designed as individually randomized, open-label trial to compare the impact of repeated (every 4 months) anthelmintic treatment versus annual treatment on the incidence of clinical malaria and the prevalence and density of malaria parasitemia among school children. A placebo-controlled trial was considered unethical because of the ongoing national school-based deworming program [25]. Although the study was open label, community health workers undertaking the malaria surveillance and laboratory technicians involved in parasitological diagnosis were blinded to treatment allocation. The primary outcome was incidence of clinical malaria assessed by 13 months of weekly active-case surveillance. Secondary outcomes were prevalence and density of *Plasmodium* species infection, assessed through cross-sectional surveys conducted at 3, 7, 11, and 15 months.

Written informed consent was obtained from a parent or guardian, and assent was sought from children before enrollment into the study. A questionnaire was administered to parents and guardians to collect information on household socioeconomic characteristics, children's use of malaria prevention measures, and recent history of deworming. The study was approved by the Kenya Medical Research Institute and National Ethics Review Committee (SSC No.2242), the London School of Hygiene and Tropical Medicine Ethics Committee (6210), and the Makerere School of Public Health Institutional Review Board (IRB00005876).

### Study Participants

Rural schools in Bumula District that were accessible year-round were purposively selected with the assistance of district officials. Initially, 30 schools were screened in January 2013 to identify schools with highest prevalence of STH infection. Subsequently, 23 schools with an STH prevalence >20% were included in the study. All children in participating schools for whom informed consent was provided were screened for STH infection, using the Kato-Katz method. To maximize the potential immunological impact of worms among participants, we initially recruited children with detectable infection with *A. lumbricoides*, *Trichuris trichiura*, and/or hookworms into the main study (n = 1505). In vitro studies have shown inhibition of *P. falciparum* by benzimidazoles [26], and a previous randomized, placebo-controlled trial suggested a possible indirect effect of albendazole treatment on clinical malaria and malaria parasitemia among preschool children [27] Therefore, to understand the impact of albendazole among children uninfected with STH, we additionally recruited 841 randomly selected uninfected children. Exclusion criteria were signs of severe malaria [28], age >15 years, STH negativity, and suspected sickle-cell trait. Recruitment was done once and closed after the baseline survey.

### Study Interventions and Randomization

Enrolled children were randomly assigned to receive either a single dose of 400 mg of albendazole (Zentel; GlaxoSmithKline South Africa, Cape Town) every 4 months for 12 months or a single dose of 400 mg of albendazole at month 0 and a single 250 mg dose of vitamin C (Cosmos, Nairobi) at 4, 8, and 12 months. Allocation to treatment group was randomized using computer-generated randomization by an independent statistician. All drugs were administered under direct observation by study nurses who were not involved in other study activities. Albendazole and vitamin C tablets were received with water. In case of vomiting within the first 30 minutes, treatment was repeated; no vomiting occurred after the second administration.

### Sample Size Calculation

The study was designed to evaluate whether the rate of clinical malaria was equivalent among the treatment groups (repeated anthelmintic treatment vs annual anthelmintic treatment), and as such sample sizes were calculated on the basis of equivalence [29]. Treatment groups were assumed to be equivalent if the difference in malaria incidence rate between groups fell between a predefined margin of 20% (−0.08 to 0.08 malaria episodes/person-year), a difference considered to represent an important public health impact. To establish equivalence within this range, assuming a malaria incidence of 0.4 episodes/year [30], with 18 months of follow-up, 80% power, and a 2-sided 95% confidence interval (CI), the sample size would be 665 children per group. To allow for loss to follow-up (20%), 753 STH-infected children would need to be enrolled in each treatment group in the main trial. For the secondary outcome, prevalence of malaria parasitemia, the proposed sample size would provide 80% power with a 2-sided 95% CI to assume equivalence between treatment groups if the difference in the malaria parasitemia between treatment groups fell within a 20% margin of −8.3% to 8.3%, given an expected malaria parasitemia prevalence of 32% (a conservative estimate) [21].

Recruitment was delayed in 2012 and follow-up curtailed in 2013 by unpredictable nationwide teacher strikes. Therefore, faced with budget and time constraints, we conducted active case surveillance for only 13 months instead of the planned 18 months. Active case detection commenced at the beginning of the next school term after the teacher strike (September 2013) and was terminated at 13 months (October 2014). With this follow-up period, a sample size of 909 children in each treatment group would be required to maintain adequate power. To account for the shortened period of follow-up, we included both infected (n = 1484) and uninfected (n = 829) children in the final analyses. This was justified on the basis of 3 reasons. First, only 61 children (9.7%) uninfected at baseline remained uninfected throughout the study. Second, baseline characteristics were generally similar among both sets of children, with exception that infected children were more likely to be male and parasitemic (Supplementary Table 1). Third, sensitivity analysis conducted including only infected children revealed no change in the direction or magnitude of results (Supplementary Tables 3 and 4).

### Procedures

The primary outcome, incidence of clinical malaria, was assessed through active case detection conducted on a weekly basis through school visits. Children absent from school were followed up at home. Axillary temperature was measured using a digital thermometer. Children with documented fever (temperature, ≥37.5°C) or who reported fever or other signs of malaria within the past 24 hours were asked to provide a blood sample by finger prick, which was used to perform a malaria rapid diagnostic test (Bioline Malaria Ag P.f/Pan, BD Biosciences, San Diego, California) and to prepare thick and thin blood smears. Any child with a diagnosis of uncomplicated clinical malaria (based on the rapid diagnostic test result) was treated with a 6-dose regimen of 20 mg artemether/120 mg lumefantrine in accordance with national guidelines.

Secondary outcomes, prevalence and density of *Plasmodium* infection, were assessed by expert microscopy during cross-sectional surveys at 0, 3, 7, 11, and 15 months. Blood smears were stained with 2% Giemsa (pH 7.2) for 45 minutes. Parasite density was defined as the number of *Plasmodium* parasites per microliter of blood, counted against 200 leukocytes and with the assumption of a leukocyte count of 8000 leukocytes/µL. If <10 asexual parasites were detected in the first 200 leukocytes, counting was continued against 500 leukocytes. A blood smear finding was considered negative when the examination of 200 high-power fields failed to reveal asexual parasites. Thin smears were used for species identification. *P. falciparum* was the only species detected. The hemoglobin concentration was assessed using a HemoCue hemoglobin photometer (Hb 201+, Ångelholm, Sweden). During the surveys conducted at 7, 11, and 15 months, children were asked to provide a stool sample, which was examined in duplicate for the presence and number of helminth ova by the Kato-Katz technique.

### Statistical Analysis

Analysis was conducted on an intention-to-treat basis (including all children randomized and entered into active case surveillance), with additional analysis conducted on a per-protocol basis (including all children who received all 4 treatment rounds). Data were analyzed using Stata, version 13 (Statacorp, College Station, Texas).

Summary statistics were calculated for all baseline data. Anthropometric indices—*z* scores of height for age (HAZ), weight for age (WAZ), and body mass index for age (BMIZ)—were calculated using the AnthroPlus software for children aged 5–19 years [31], assuming a midpoint age for each child. Weight for age was calculated only for the children aged 5–10 years. Children were classified as stunted, underweight, and thin if their HAZ, WAZ, and BMIZ, respectively, were less than −2 SDs from the reference medium. Anemia was defined using age- and sex-specific World Health Organization thresholds adjusted on the basis of altitude [32].

Clinical malaria was defined as the presence of asexual *Plasmodium* species parasitemia (as determined by microscopy) and either an axillary temperature of >37.5°C or a reported history of fever or other signs of malaria during the preceding 24 hours. An alternative case definition that used a parasite density cutoff of >2500 parasites/µL was also used [33]. Children were considered at risk from their date of entry into the study until experiencing an episode of clinical malaria completing follow-up at 13 months. Children who had documented or reported clinical malaria or who were known to have received medical attention from any source other than the survey team were censored for 28 days. Children who were absent at school for ≥10 days were censored for the time of absence. Rates in each treatment group were calculated as the number of events divided by the number of person-years at risk, and relative differences in rate ratios were determined using survival analysis functions in Stata. Survival analysis for clinical malaria up to 13 months was based on Kaplan–Meier curves. The prevalences of STH and *Plasmodium* infection, together with their 95% CIs, were calculated using exact binomial analysis. To allow for overdispersion of egg counts, arithmetic mean numbers of eggs per gram of feces, with their 95% CIs, were estimated using negative binomial regression, taking into account clustering by school. The differences in the prevalence of malaria parasitemia between treatment groups at 0, 3, 7, 12, and 15 months were determined using the binomial test for differences in proportions.

Equivalence between treatment groups can be stated if the 95% CIs of the relative difference in malaria incidence and parasitemia lie within the predetermined margins of equivalence of −0.08 to 0.08 episodes/person-year for clinical malaria and −8.3% to 8.3% for prevalence of malaria parasitemia. Sensitivity analyses were performed to examine the intervention effect, excluding children who were uninfected at baseline.

## RESULTS

### Trial Profile and Baseline Characteristics

**Figure 1.**
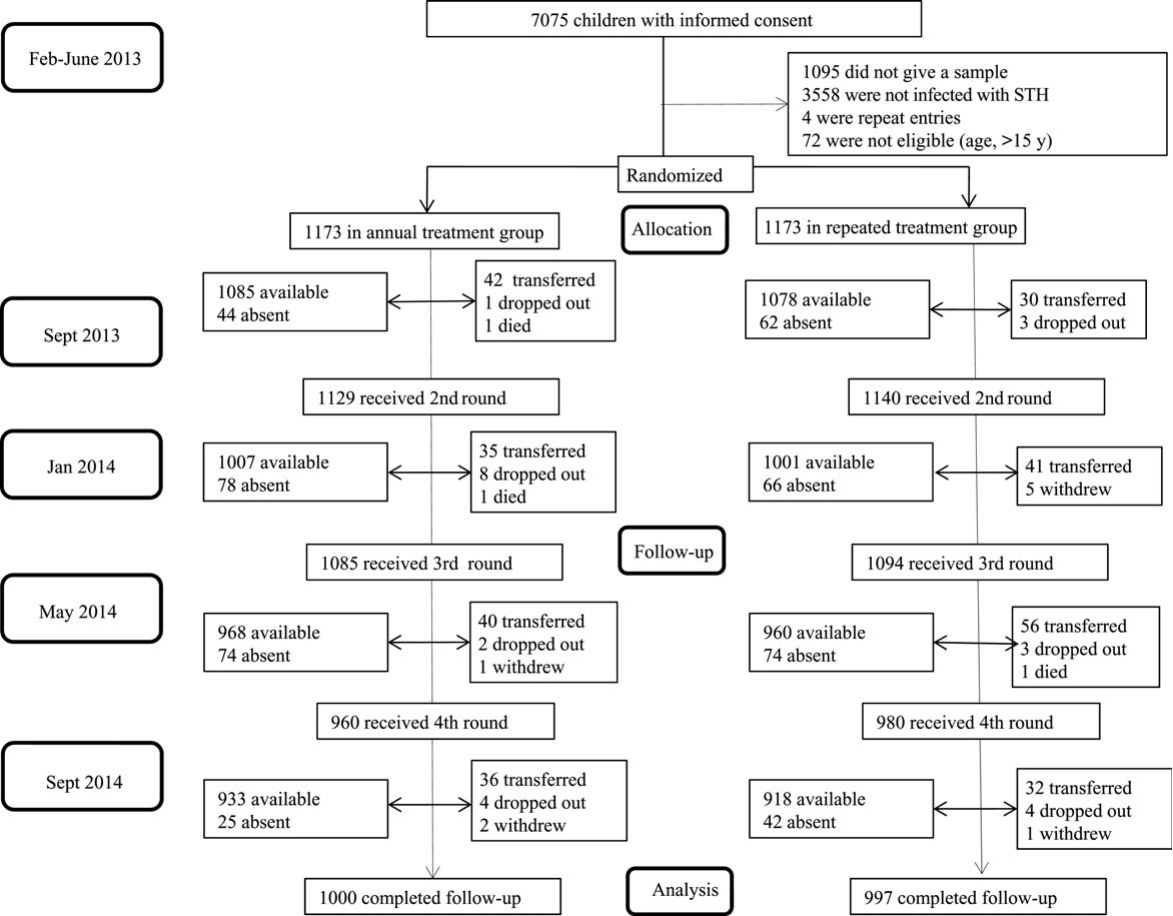
Trial profile. Children whose parents withdrew consent and children who refused to be part of the study were categorized as “withdrew”; children who stopped coming to school were categorized as “dropped out”; and children who moved to schools not in the study were categorized as “transferred.” Abbreviation: STH, soil-transmitted helminths.

Between February and June 2013, 7075 children aged 5–18 years were screened; 1505 infected with at least 1 STH species were recruited into the trial. A further 841 randomly selected children without STH infection were also recruited. In total, 2346 children were included in the baseline survey and randomized to receive either repeated or annual treatment. Baseline characteristics were comparable between the 2 treatment groups (Table 1). Between the baseline survey and the start of active case surveillance, 33 children were lost to follow-up, leaving 2313 children. During the course of the trial, 1937 children (82.5%) received all 4 doses of albendazole or albendazole/vitamin C; 240 (10.2%) received 3 doses, 97 (4.1%) received 2 doses, and 72 (3.1%) received only 1 dose. The trial profile (Figure 1) shows that 1000 children (85.3%) in the repeated treatment group and 997 (85.0%) in the annual treatment group completed the full 13 months of active case surveillance follow-up.

**Table 1.** Baseline Characteristics of Children

Characteristic	Study Group	
	Annual Treatment (n = 1173)	Repeated Treatment (n = 1173)
Male sex	52.7 (618/1173)	52.3 (614/1173)
Age, y, mean ± SD	10.5 ± 2.5	10.4 ± 2.5
Body temperature, °C, mean ± SD	36.6 ± 0.7	36.5 ± 1.1
*z* score less than −2 SD below median reference value		
Weight for age	2.9 (34/1173)	3.2 (37/1173)
Height for age	25.8 (303/1173)	24.7 (290/1173)
Body mass index for age	10.8 (127/1173)	10.1 (118/1173)
Malaria parasitemia^a^	48.4 (546/1129)	48.3 (549/1136)
Parasitemia level, parasites/µL, mean (95% CI)	1626 (1104–2393)	2143 (1571–2925)
STH prevalence		
Hookworm	38.3 (449/1173)	38.1 (443/1173)
*A. lumbricoides*	35.0 (411/1173)	36.6 (429/1173)
*T. trichiura*	1.0 (12/1173)	0.6 (7/1173)
Any STH infection	64.2 (753/1173)	64.1 (752/1173)
STH intensity, eggs/g of feces, mean (95% CI)		
Hookworm	68 (44–106)	119 (75–191)
*A. lumbricoides*	1979 (1509–2596)	1693 (1263–2269)
Coinfection		
Hookworm and *A. lumbricoides*	10.0 (117/1173)	11.3 (131/1173)
Hookworm and *P. falciparum*	21.3 (239/1129)	20.1 (227/1136)
*A. lumbricoides* and *P. falciparum*	18.1 (204/1129)	18.1 (204/1136)
Hemoglobin level, g/dL, mean (standard error)	12.3 ± 1.3	12.3 ± 1.4
Anemia	36.8 (402/1093)	39.2 (432/1103)
Slept under bed net previous night	49.0 (854/1100)	51.0 (888/1113)
Education level of household head		
None or incomplete primary	49.6 (623/1090)	50.1 (632/1095)
Above primary school	50.2 (467/1090)	49.8 (463/1095)

Data are % (proportion) of children, unless otherwise indicated.

Abbreviations: *A. lumbricoides*, *Ascaris lumbricoides*; CI, confidence interval; *P. falciparum*, *Plasmodium falciparum*; SD, standard deviation; STH, soil-transmitted helminth; *T. trichiura*, *Trichuris trichiura*.

^a^ Determined by microscopy.

### Follow-up Data

**Figure 2.**
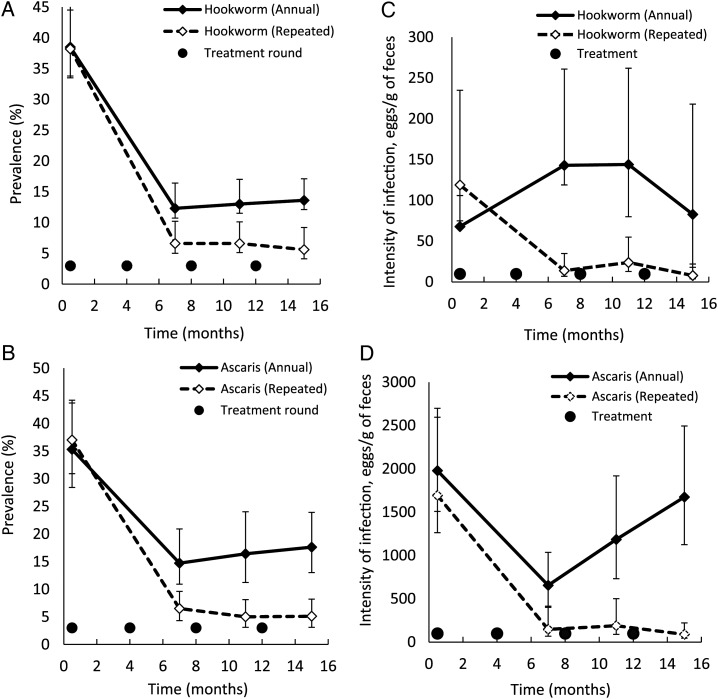
Prevalence and intensity of hookworm (*A* and *C*) and *Ascaris lumbricoides* (*B* and *D*) during the 4 cross-sectional surveys. Whiskers denote 95% confidence intervals. The point of origin x-axis is shifted right to make error bars visible.

Table 2 and Figure 2 present the effect of repeated and annual treatment on *A. lumbricoides* and hookworm prevalence over time. *T. trichiura* was very rare among study participants. Repeated treatment markedly reduced the prevalence of STH infection to <10% after 7 months and kept levels low (<6%) at 15 months of follow-up. In contrast, annual treatment only reduced the prevalence of *A. lumbricoides* to 14.7% and the prevalence of hookworm to 13.0% at 7 months of follow-up, and thereafter, at 11 and 15 months, infection levels rose slightly, to 24.8% and 26.4%, respectively. Of the 1505 children who were infected with any STH species at baseline, 1141 (75.8%) were followed up, and 257 (22.5%) remained infected at 15 months. Of the 841 children uninfected at baseline, 631 (75.0%) were followed up, and 61 (9.7%) remained uninfected at 15 months.

**Table 2.** Prevalence and Intensity of Hookworm, *Ascaris lumbricoides*, and Any Soil-Transmitted Helminth (STH) Infection During the Cross-sectional Surveys

Month (Survey Time), STH Species	Children, No.	Prevalence, % (95% CI)		*P Value*
		Annual Treatment	Repeated Treatment	
0 (Feb–June 2013)	2346			
Hookworm		38.3 (33.1–44.3)	37.7 (33.1–43.1)	.799
*A. lumbricoides*		35.0 (28.2 43.4)	36.5 (30.6–43.6)	.438
Any STH		63.8 (59.4–68.6)	63.4 (60.3–66.7)	.830
7 (Jan 2014)	1969			
Hookworm		12.3 (9.2–16.4)	6.6 (5.0–8.6)	.001
* A. lumbricoides*		14.7 (10.9–19.9)	6.4 (4.3–9.5)	<.001
Any STH		23.8 (19.4–29.0)	11.7 (9.6–14.5)	<.001
11 (May 2014)	1870			
Hookworm		13.0 (10.0–17.0)	6.6 (5.1–8.6)	<.001
* A. lumbricoides*		16.3 (11.1–24.0)	5.0 (3.1–8.1)	<.001
Any STH		24.6 (19.4–31.3)	10.2 (7.9–13.1)	<.001
15 (Sept 2014)	1772			
Hookworm		13.6 (10.8–17.1)	5.6 (4.1–7.7)	<.001
*A. lumbricoides*		17.6 (13.0–23.9)	5.0 (3.1–8.1)	<.001
Any STH		26.5 (21.8–32.2)	9.6 (7.4–12.4)	<.001
		Intensity of Infection, Eggs/g (95% CI)		
		Annual Treatment	Repeated Treatment	
0 (Feb–June 2013)	2346			
Hookworm		68 (43–105)	118 (75–189)	.004
* A. lumbricoides*		1966 (1500–2596)	1673 (1249–2240)	.093
7 (Jan 2014)	1969			
Hookworm		143 (79–261)	14 (7–28)	<.001
*A. lumbricoides*		656 (415–1037)	148 (68–321)	<.001
11 (May 2014)	1870			
Hookworm		144 (79–262)	24 (13–44)	<.001
* A. lumbricoides*		1185 (732–1919)	190 (89–399)	<.001
15 (Sept 2014)	1772			
Hookworm		83 (32–218)	8 (4–18)	<.001
* A. lumbricoides*		1672 (1121–2491)	89 (45–176)	<.001

Abbreviation: CI, confidence interval.

**Figure 3.**
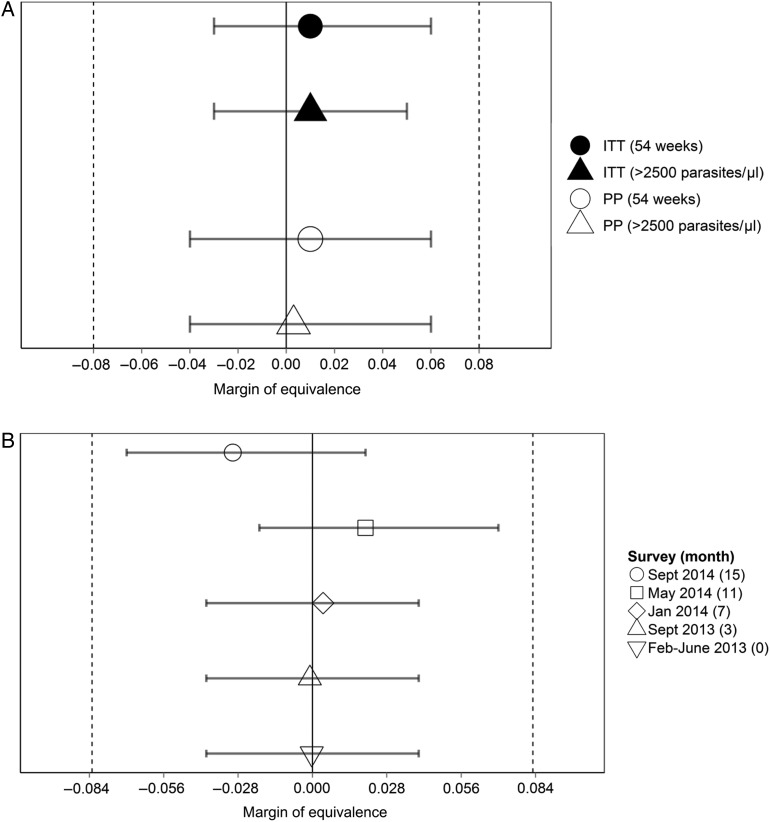
Absolute incidence rate difference over 13 months of follow-up between treatment groups (*A*) and the absolute difference in the prevalence of malaria parasitemia between the treatment groups during 5 cross-sectional surveys (*B*). Whiskers denote 2-sided 95% confidence intervals, vertical dashed lines denote zones of predefined equivalence, and solid lines denote null scales. Abbreviations: ITT, intention-to-treat analysis; PP, per-protocol analysis.

**Figure 4.**
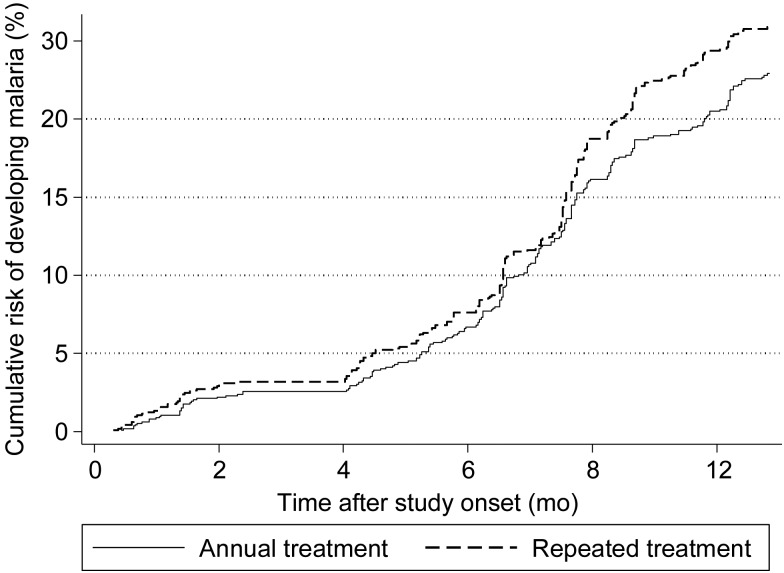
Cumulative risk of malaria over 13 months of follow-up, by treatment group.

During the 13 months of active case detection, 606 incident cases of malaria occurred, with 405 children having only 1 incident case and 93 having ≥2 cases. The incidence of malaria was 0.27 episodes/person-year in the repeated treatment group and 0.26 episodes/person-year in the annual treatment group, a rate difference of 0.01 (95% CI, −.03 to .06), based on intention-to-treat analysis. Figure 3*A* presents comparisons of incidence rates among treatment groups and shows that CIs included 0 and were within the predefined margin of equivalence (−0.08 to 0.08 malaria episodes/person-year) for intention-to-treat and per-protocol analyses. Similarly, by using a malaria case definition based on an increased parasite density cutoff, the results showed equivalence (Figure 3*A*). Figure 4 presents the Kaplan–Meier survival curves for clinical malaria up to 13 months and shows similar curves for each treatment group. Although there was a slight diversion after 8 months, the difference was small. The absolute prevalence difference in malaria parasitemia and the associated 95% CI at each of the 5 cross-sectional surveys fell within the predefined margin of equivalence (Figure 3*B* and Table 3). The density of parasitemia was similar in each treatment group (Table 3). Sensitivity analysis showed comparable results when analysis was restricted to children with detectable STH infection (Supplementary Tables 2–4).

**Table 3.** Prevalence and Density of Malaria Parasitemia at Each Cross-sectional Survey

Month (Survey Time)	Season	Children, No.	Prevalence, % (95% CI)		Proportion Difference (95% CI)
			Annual Treatment	Repeated Treatment	
0 (Feb–June 2013)	Dry	2265	48.3 (42.7–54.7)	48.4 (43.7–53.6)	0.000 (−.04 to .04)
3 (Sept 2013)	Wet	2163	41.5 (36.2–47.6)	41.8 (36.8–47.4)	−0.001 (−.04 to .04)
7 (Jan 2014)	Dry	2008	32.3 (26.4–39.6)	31.9 (26.0–39.1)	0.004 (−.04 to .04)
11 (May 2014)	Wet	1928	43.7 (38.3–50.0)	41.5 (37.2–46.3)	0.02 (−.02 to .07)
15 (Sept 2014)	Wet	1851	42.9 (37.8–48.8)	45.3 (40.4–50.8)	−0.03 (−.07 to .02)
			Density, Parasites/µL (95% CI)		Mean Difference (95% CI)
			Annual Treatment	Repeated Treatment	
0 (Feb–June 2013)	Dry	2265	1626 (1105–2393)	2144 (1571–2926)	−497 (−1417 to 424)
3 (Sept 2013)	Wet	2163	460 (370–573)	1149 (452–2924)	−683 (−1774 to 408)
7 (Jan 2014)	Dry	2008	602 (442–821)	507 (366–704)	94 (145–335)
11 (May 2014)	Wet	1928	1210 (891–1645)	1345 (954–1896)	−120 (−613 to 371)
15 (Sept 2014)	Wet	1851	577 (475–703)	670 (493–912)	−92 (−304 to 121)

Abbreviation: CI, confidence interval.

## DISCUSSION

The results of our trial show that 4 rounds of albendazole at 4 monthly intervals did not increase or decrease the incidence of clinical malaria or malaria parasitemia, compared with a single round of treatment, based on our predefined margins of equivalence (±0.08 cases/person-year and ±8.3%, respectively). Our study has a number of strengths, including a randomized design, a prespecified sample size, a specific case definition of malaria, and a high (85%) follow-up rate. A previous study in Nigeria found that anthelmintic treatment every 4 months for 14 months resulted in an increase in *Plasmodium* infection in the dry season, compared with findings in the placebo group, but this difference was found to be nonsignificant, partly because of the very low follow-up rate (26%) [17]. A placebo-controlled trial in Uganda involving a birth cohort found that quarterly albendazole treatment reduced the malaria incidence among children aged <5 years, with the strongest effects among children aged 15–24 months [27]. However, this study was unable to determine whether this observation was due to removal of worms from children or to the direct inhibitory effect of albendazole on malaria parasites. A recent study in Tanzania found that repeated anthelmintic treatment with praziquantel and albendazole 4 times/year did not change the incidence of malaria or the prevalence of *Plasmodium* among children, compared with findings associated with annual treatment [19]. This study did not include an explicit sample size calculation, recruiting some 100 children per school, and relied on schoolteachers to identify clinical cases of malaria over 2 years of follow-up. In Indonesia, an adequately powered and rigorous cluster-randomized trial reported no effect of repeated treatment with albendazole on the prevalence of malaria parasitemia among school children. Combined, these previous studies and our study suggest that repeated school-based deworming has no indirect effects on the risk of clinical malaria and malaria parasitemia among school children.

There are a number of study limitations. The study was partially unblinded; participants were aware of treatment allocation, but assessors of main outcomes were blind. Second, our study reflects parasitemia based on expert microscopy findings, which underestimates infection, compared with molecular methods [34]. Third, children may have sought treatment from other sources and may therefore have been missed by our active case surveillance, but our randomized design should have minimized bias between groups. Fourth, the ethical obligation to provide at least annual deworming, the standard of care in Kenya, meant that we were unable to include an untreated control group. We expected 4 rounds of treatment to completely clear infection among children in the repeated treatment group. At 15 months of follow-up, however, 9.7% of children still harbored STH infections, compared with 26.5% in the annual treatment group. As such, the low levels of infection might explain the lack of difference between the treatment groups. Given that the strength of immunological responses against helminths depend partly on infection intensity [8, 35], another potential contributing factor to the lack of difference are the relatively low intensities found in western Kenya, compared with other settings [16, 17, 36]. However, low levels of infection are increasingly becoming the norm as countries implement national deworming programs, and as such our results have relevance for many settings in sub-Saharan Africa.

In areas of high malaria transmission, the main burden of malaria is among young children, but the risk of coinfection is low because of low levels of helminth infections in this age group [37]. It would nonetheless be important to investigate the impact of deworming on the risk of malaria among young children, because an effect has been previously documented [27] and because young children receive deworming, either as part of school-based deworming programs or during child health days. In contrast to their younger siblings, school-age children living in areas of high malaria transmission have generally acquired immunity to malaria [38] and therefore tend to experience less morbidity from malaria. However, although the burden of malaria may be low, it is not insignificant [39]. In this study, 1 in 5 children experienced clinical malaria, and 30%–50% of children, depending on the season during which the survey was performed, were infected with malaria parasites. Future analysis will investigate risk factors for malaria morbidity and association with anemia.

An estimated 81.6 million school-aged children living in sub-Saharan Africa benefitted from mass treatment with albendazole or mebendazole in 2013 [40]. The results from our study, together with other work, show that repeated anthelmintic treatment does not increase or decrease the rate of clinical malaria or the risk of malaria parasitemia. These findings offer evidence for use in the planning of school-based deworming in sub-Saharan Africa and show that the scaling up of deworming is unlikely to have adverse consequences for malaria among school-aged children.

## Supplementary Data

Supplementary materials are available at http://jid.oxfordjournals.org. Consisting of data provided by the author to benefit the reader, the posted materials are not copyedited and are the sole responsibility of the author, so questions or comments should be addressed to the author.

Supplementary Data
