# Supplementary material for: Effect of Repeated Anthelminthic Treatment on Malaria in School Children in Kenya: A Randomized, Open-Label, Equivalence Trial
Source: J Infect Dis. 2015 Jul 13;213(2):266–75. doi: 10.1093/infdis/jiv382 (PMC4690148; doi:10.1093/infdis/jiv382)
Supplement: Supplementary Data [file supp_jiv382_jiv382supp.doc]

# CONSORT Statement 2006 - Checklist for Non-inferiority and Equivalence Trials
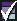


**Items to include when reporting a non-inferiority or equivalence randomized trial**

| ***PAPER SECTION* And topic** | Item | **Descriptor** | **Section and paragraph (P) No** |
| --- | --- | --- | --- |
| ***TITLE & ABSTRACT*** | | | |
|  | 1a | How participants were allocated to interventions | Title |
|  | 1b | Structured summary of trials, design, methods, results, and conclusions (for specific guidance see the CONSORT for abstracts^7,8^) | Abstract |
| **INTRODUCTION** | | | |
| **Background** | 2a | Scientific background and explanation of rationale,  *Including the rationale for using a non-inferiority or equivalence design.* | Background- Paragraph 1 and 2 |
| **Objectives** | 2b | Specific objectives or hypotheses | Background- Paragraph 2 |
| **METHODS** | | | |
| **Trial design** | 3a | Descriptions of the trial design (such as parallel, factorial), including allocation ratio | Materials and methods- Study design paragraph 1 & 2  Figure 1 |
|  | 3b | Important changes to methods after trial commencement (such eligibility criteria), with reasons | n/a |
| **Participants** | 4a | Eligibility criteria for participants *(detailing whether participants in the non-inferiority or equivalence trial are similar to those in any trial(s) that established efficacy of the reference treatment)* | Materials and Methods- Study participants |
|  | 4b | settings and locations where the data were collected | Materials and Methods- Study design, study participants |
| **Interventions** | 5 | Precise details of the interventions intended for each group and how and when they were actually administered. | Materials and Methods- Study intervention and randomization |
| **Outcomes** | 6a | Clearly defined prespecified primary and secondary outcome measures methods used to enhance the quality of measurements (*e.g.*, multiple observations, training of assessors). | Material and Methods- Study design paragraph one |
|  | 6b | Any changes to trial outcomes after the trail commenced, with reasons | n/a |
| **Sample size** | 7a | How sample size was determined (*and whether related to a non-inferiority or equivalence hypothesis*). | Materials and Methods- Sample size calculation |
|  | 7b | When applicable, explanation of any interim analyses and stopping rules | n/a |
| **Randomization** |  |  |  |
| **Sequence generation** | 8a | Method used to generate the random allocation sequence, including details of any restrictions (*e.g*., blocking, stratification) | Materials and Methods- Study intervention and randomization |
|  | 8b | Type of randomization; details of any restrictions( such as blocking and block size) | Materials and Methods- Study design |
| **Allocation concealment** | 9 | Method used to implement the random allocation sequence (*e.g*., numbered containers or central telephone), clarifying whether the sequence was concealed until interventions were assigned. | Materials and Methods- Study intervention and randomization |
| **Implementation** | 10 | Who generated the allocation sequence, who enrolled participants, and who assigned participants to their groups? | Materials and Methods- Study intervention and randomization |
| **Blinding (masking)** | 11 | Whether or not participants, those administering the interventions, and those assessing the outcomes were blinded to group assignment. If done, how the success of blinding was evaluated. | Abstract  Materials and Methods-  Study design paragraph 1&  Study intervention and randomization  Discussion- Paragraph 3 |
| **Statistical methods** | 12a | Statistical methods used to compare groups for primary outcome | Materials and Methods- Statistical analysis |
|  | 12b | Methods for additional analyses, such as subgroup analyses and adjusted analyses | Materials and Method- Statistical analysis |
| **RESULTS** |  |  |  |
| **Participant flow**(a diagram is strongly recommended) | 13a | For each group report the numbers of participants randomly assigned, received intended treatment, completing the study protocol, and analyzed for the primary outcome. | Figure 1 |
|  | 13b | For each group, losses and exclusion after randomization | Figure 1 |
| **Recruitment** | 14a | Dates defining the periods of recruitment and follow-up. | Materials and Methods-Procedures paragraph 1  Figure1 |
|  | 14b | Dates defining the periods of recruitment and follow-up | Results- Paragraph 1  Figure 1 |
| **Baseline data** | 15 | Baseline demographic and clinical characteristics of each group. | Table 1, Supplementary Table 1 |
| **Numbers analyzed** | 16 | Number of participants (denominator) in each group included in each analysis and whether the analysis was *“intention-to-treat”* *and/or* *alternative analyses were conducted*. State the results in absolute numbers when feasible (*e.g*., 10/20, not 50%). | Results-Paragraph1  Table 2 |
| **Outcomes and estimation** | 17a | For each primary and secondary outcome, a summary of results for each group, and the estimated effect size and its precision (*e.g.*, 95% confidence interval). | Table 3, Figure 3, Figure 4 |
|  | 17b | For binary outcomes, presented of both absolute and relative effect size is recommended | Table 3 |
| **Ancillary analyses** | 18 | Address multiplicity by reporting any other analyses performed, including subgroup analyses and adjusted analyses, indicating those pre-specified and those exploratory. | Supplementary Tables 1-4 |
| **Harms** | 19 | All important harms or unintended effects in each group (for specific guidance see CONSORT for harms^26^ ) | n/a |
| **DISCUSSION** |  |  |  |
| **Interpretation** | 20 | Trials limitations, addressing sources of potential bias, imprecision, and if relevant, multiplicity of analyses | Discussion-Paragraph 3 |
| **Generalizability** | 21 | Generalizability (external validity) of the trial findings. | Abstract  Discussion-Paragraph 3 &4 |
| **Interpretation** | 22 | Interpretation consistent with results, balancing benefits and harms and considering other relevant evidence. (interpret results in relation to the noninferiority hypothesis | Abstract-Conclusion  Discussion- Paragraph 2, 3 & 4 |
| **Registration** | 23 | Registration number name and trial registry | Abstract |
| **Protocol** | 24 | Where the full trial protocol can be accessed, if available | n/a |
| **Funding** | 25 | Sources of funding and other support (such as supply of drugs), roles of funders | Financial Support |

**www.consort-statement.org**
