# Supplementary material for: Effect of Repeated Anthelminthic Treatment on Malaria in School Children in Kenya: A Randomized, Open-Label, Equivalence Trial
Source: J Infect Dis. 2015 Jul 13;213(2):266–75. doi: 10.1093/infdis/jiv382 (PMC4690148; doi:10.1093/infdis/jiv382)
Supplement: Supplementary Data [file supp_jiv382_jiv382supp_table1.docx]

**Supplementary Table 1. Baseline characteristics of children infected with any soil- transmitted helminth species versus children uninfected at recruitment.**

| **Characteristic^1^** | **Infected**  **(N=1505)** | **Uninfected**  **(N=841)** |
| --- | --- | --- |
| Sex, male, % (n/N) | 54.0 (813/1,505) | 49.8 (419/841) |
| Age, years, mean (SD) | 10.5 (2.4) | 10.4 (2.5) |
| Mean body temperature, mean^o^C (SD) | 36.6 (0.9) | 36.5 (1.1) |
| WAZ<-2 SD below median reference value | 3.0 (45/1,505) | 3.1 (26/841) |
| HAZ <-2 SD below median reference value | 26.9 (405/1,505) | 22.5 (188/841) |
| BMIZ <-2 SD below median reference value | 12.3 (181/1,505) | 7.6 (64/841) |
| Malaria parasitaemia | 51.0 (744/1,460) | 43.4 (351/805) |
| Parasitaemia, parasites/μL, mean (95% CI) | 1825 (1,351-2,465) | 1995 (1,304-3,034) |
| Hemoglobin, g/dL, mean (SD) | 12.3 (1.3) | 12.3 (1.4) |
| Anemia | 38.7 (545/1408) | 36.9 (289/788) |
| Slept under a bednet previous night | 77.5 (1,110/1,430) | 80.9 (632/781) |
| Education level of household head |  |  |
| None or incomplete primary | 58.2 (825/1,418) | 56.1 (430/767) |
| Above primary school | 41.8 (593/1,418) | 43.9 (337/767) |

Abbreviations: SD, standard deviation; WAZ, weight-for-age z-score; HAZ, height-for-age z-score; BMIZ, body mass index z-score; CI, confidence interval

^1^ Data are proportions (N/n), unless otherwise stated.
