# Supplementary material for: Effect of Repeated Anthelminthic Treatment on Malaria in School Children in Kenya: A Randomized, Open-Label, Equivalence Trial
Source: J Infect Dis. 2015 Jul 13;213(2):266–75. doi: 10.1093/infdis/jiv382 (PMC4690148; doi:10.1093/infdis/jiv382)
Supplement: Supplementary Data [file supp_jiv382_jiv382supp_table2.docx]

**Supplementary Table 2. Baseline characteristics among children who were infected with any STH at recruitment.**

|  | **Study group** | |
| --- | --- | --- |
| **Characteristic^1^** | **Annual treatment**  **(N=753)** | **Repeated treatment**  **(N=752)** |
| Sex, male | 55.4 (417/753) | 52.7 (396/752) |
| Age, years, mean (SD) | 10.4 (2.5) | 10.4 (2.5) |
| Mean body temperature, mean^o^C (SD) | 36.5 (0.8) | 36.6 (1.0) |
| WAZ<-2 SD below median reference value | 3.1 (23/753) | 2.9 (22/752) |
| HAZ <-2 SD below median reference value | 27.4 (206/753) | 26.5 (199/752) |
| BMIZ <-2 SD below median reference value | 12.9 (97/753) | 11.2 (84/752) |
| Malaria parasitaemia | 51.8 (379/731) | 50.1 (365/729) |
| Parasitaemia, parasites/μL, mean (95% CI) | 1,331 (976-1,814) | 2,320 (1,509-3,568)) |
| STH prevalence |  |  |
| Hookworm | 59.6 (449/753) | 38.1 (441/752) |
| *Ascarislumbricoides* | 54.4 (410/753) | 56.9 (428/752) |
| *Trichuristrichiura* | 1.5 (12/753) | 0.9 (7/753) |
| Any STH infection | 100 (753/753) | 100(752/752) |
| STH intensity, eggs/g faeces, mean(95%CI) |  |  |
| Hookworm | 106 (68-164) | 186 (116-297) |
| *A.lumbricoides* | 3,062 (2,421-3,871) | 2,601 (1,979-3,4206) |
| Coinfection |  |  |
| Hookworm-*A.lumbricoides* | 15.5 (117/753) | 17.3 (131/752) |
| Hookworm-*P. falciparum* | 32.7 (239/731) | 30.9 (225/729) |
| *A.lumbricoides*-*P-falciparum* | 27.8 (203/731) | 27.9 (203/729) |
| Hemoglobin, g/dL, mean (SD) | 12.3 (1.3) | 12.3 (1.4) |
| Anemia | 37.6 (264/702) | 39.8 (281/706) |
| Slept under a bednet previous night | 79.2 (542/712) | 78.9 (568/720) |
| Education level of household head |  |  |
| None or incomplete primary | 58.1 (412/709) | 58.2 (413/709) |
| Above primary school | 41.9 (297/709) | 42.8 (296/709) |

Abbreviations: SD, standard deviation; WAZ, weight-for-age z-score; HAZ, height-for-age z-score; BMIZ, body mass index z-score; STH, soil-transmitted helminth; CI, confidence interval

^1^ Data are proportions (N/n), unless otherwise stated.
