# Supplementary material for: Effect of Repeated Anthelminthic Treatment on Malaria in School Children in Kenya: A Randomized, Open-Label, Equivalence Trial
Source: J Infect Dis. 2015 Jul 13;213(2):266–75. doi: 10.1093/infdis/jiv382 (PMC4690148; doi:10.1093/infdis/jiv382)
Supplement: Supplementary Data [file supp_jiv382_jiv382supp_table3.docx]

**Supplementary Table 3. Prevalence of hookworm, *Ascaris lumbricoides* and any STH infection among children who were infected with any STH at recruitment during the cross-sectional surveys.**

|  |  |  | **Prevalence, % (95% CI)** | |  |
| --- | --- | --- | --- | --- | --- |
| **Month**  **(survey date)** | **N** | **STH species** | **Annual treatment** | **Repeated treatment** | **P value** |
| 0 (Feb-June 2013) | 1,505 | Hookworm | 59.9 (51.9-69.2) | 59.2 (52.9-67.5) | 0.799 |
|  |  | *A.lumbricoides* | 54.4 (46.0-67.2) | 57.4 (49.1-67.2) | 0.399 |
|  |  | Any STH | 64.3 (59.9-69.0) | 64.2 (60.9-67.6) | 0.954 |
| 7 (Jan 2014) | 1,271 | Hookworm | 15.2 (11.4-20.2) | 9.2 (7.0-12.1) | 0.006 |
|  |  | *A.lumbricoides* | 7.9 (5.4-11.5) | 19.1 (10.0-26.2) | <0.001 |
|  |  | Any STH | 29.4 (23.9-36.1) | 15.2 (12.8-18.2) | <0.001 |
| 11 (May 2014) | 1,198 | Hookworm | 17.0 (13.2-22.0) | 9.2 (6.8-12.7) | <0.001 |
|  |  | *A.lumbricoides* | 22.4 (15.6-31-8) | 6.0 (4.0-11.0) | <0.001 |
|  |  | Any STH | 32.9 (27.1-40.1) | 13.7 (10.4-18.1) | <0.001 |
| 15 (Sept 2014) | 1,141 | Hookworm | 18.3 (4.4-23.3) | 5.7 (4.2-7.8) | <0.001 |
|  |  | *A.lumbricoides* | 23.4 (17.6-30.9) | 6.2 (3.7-10.6) | <0.001 |
|  |  | Any STH | 34.6 (29.3-41.3) | 10.8 (8.4-13.8) | <0.001 |
|  |  |  | **Intensity of infection, eggs/gram (95% CI)** | |  |
| **Month**  **(survey date)** | **N** | **STH species** | **Annual treatment** | **Repeated treatment** | **P value** |
| 0 (Feb-June 2013) | 1,505 | Hookworm | 106 (68-165) | 186 (116-297) | 0.005 |
|  |  | *A.lumbricoides* | 3708 (2,432-3,894) | 2637 (2,006-3,466) | <0.001 |
| 7 (Jan 2014) | 1,271 | Hookworm | 218 (118-403) | 20 (10-43) | <0.001 |
|  |  | *A.lumbricoides* | 935 (596-1,467) | 204 (89-469) | <0.001 |
| 11 (May 2014) | 1,198 | Hookworm | 210 (113-392) | 30 (15-59) | <0.001 |
|  |  | *A.lumbricoides* | 1745 (1,102-2763) | 287 (136-608) | <0.001 |
| 15 (Sept 2014) | 1,141 | Hookworm | 127 (48-337) | 7 (3-15) | <0.001 |
|  |  | *A.lumbricoides* | 2508 (1,721-3656) | 118 (57-246) | <0.001 |

Abbreviations: STH, soil-transmitted helminth; CI, confidence interval; STH, soil-transmitted helminth
