# Supplementary material for: Effect of Repeated Anthelminthic Treatment on Malaria in School Children in Kenya: A Randomized, Open-Label, Equivalence Trial
Source: J Infect Dis. 2015 Jul 13;213(2):266–75. doi: 10.1093/infdis/jiv382 (PMC4690148; doi:10.1093/infdis/jiv382)
Supplement: Supplementary Data [file supp_jiv382_jiv382supp_table4.docx]

**Supplementary Table 4. Prevalence and density of malaria parasitaemia among children who were infected with any STH at recruitment during each cross-sectional survey.**

|  |  |  | **Prevalence, % (95% CI)** | |  |
| --- | --- | --- | --- | --- | --- |
| **Month (survey date)** | **Season** | **Number examined** | **Annual treatment** | **Repeated treatment** | **Proportion difference, (95% CI)** |
| 0 (Feb-June 2013) | Dry | 1,460 | 51.9 (45.7-59.0) | 50.1 (45.2-53.6) | 0.02 (-0.03, 0.07) |
| 3 (Sept 2013) | Wet | 1,386 | 42.6 (37.4-48.6) | 44.6 (39.8-49.9) | -0.02 (-0.07, 0.03) |
| 7 (Jan 2014) | Dry | 1,299 | 33.3 (26.5-42.0) | 34.5 (27.8-43.0) | -0.01 (-0.06, 0.04) |
| 11 (May 2014) | Wet | 1,242 | 46.2 (39.9-53.6) | 42.5 (37.2-48.5) | 0.04 (-0.02, 0.09) |
| 15 (Sept 2014) | Wet | 1,187 | 44.8 (39.2-51.2) | 47.6 (41.7-54.2) | -0.03 (-0.09, 0.03) |
|  |  |  | Density, parasites/μL (95% CI) | |  |
|  |  |  | **Annual treatment** | **Repeated treatment** | **Proportion difference, (95% CI)** |
| 0 (Feb-June 2013) | Dry | 1,460 | 1,331 (976-1,814) | 2,321 (1,509-3,568) | -980 (-2122,163) |
| 3 (Sept 2013) | Wet | 1,386 | 471 (378-588) | 1,480 (485-4,518) | -999 (-2685, 687) |
| 7 (Jan 2014) | Dry | 1,299 | 642 (419-985) | 506 (348-736) | 137 (-183, 457) |
| 11 (May 2014) | Wet | 1,242 | 1,323 (897-1,954) | 1,128 (764-1,665) | 195 (-346, 736) |
| 15 (Sept 2014) | Wet | 1,187 | 545 (422-707) | 630 (467-852) | -84 (-303, 1341) |

Abbreviations: CI, confidence interval: STH, soil-transmitted helminth
